# Comparison of Calibration Approaches in Laser-Induced Breakdown Spectroscopy for Proximal Soil Sensing in Precision Agriculture

**DOI:** 10.3390/s19235244

**Published:** 2019-11-28

**Authors:** Daniel Riebe, Alexander Erler, Pia Brinkmann, Toralf Beitz, Hans-Gerd Löhmannsröben, Robin Gebbers

**Affiliations:** 1 Physical Chemistry, University of Potsdam, Karl-Liebknecht-Str. 24-25, 14476 Potsdam, Germany; riebe@uni-potsdam.de (D.R.);; 2 Leibniz Institute for Agricultural Engineering and Bioeconomy (ATB), Max-Eyth-Allee 100, 14469 Potsdam, Germany

**Keywords:** laser-induced breakdown spectroscopy, LIBS, proximal soil sensing, soil nutrients, elemental composition

## Abstract

The lack of soil data, which are relevant, reliable, affordable, immediately available, and sufficiently detailed, is still a significant challenge in precision agriculture. A promising technology for the spatial assessment of the distribution of chemical elements within fields, without sample preparation is laser-induced breakdown spectroscopy (LIBS). Its advantages are contrasted by a strong matrix dependence of the LIBS signal which necessitates careful data evaluation. In this work, different calibration approaches for soil LIBS data are presented. The data were obtained from 139 soil samples collected on two neighboring agricultural fields in a quaternary landscape of northeast Germany with very variable soils. Reference analysis was carried out by inductively coupled plasma optical emission spectroscopy after wet digestion. The major nutrients Ca and Mg and the minor nutrient Fe were investigated. Three calibration strategies were compared. The first method was based on univariate calibration by standard addition using just one soil sample and applying the derived calibration model to the LIBS data of both fields. The second univariate model derived the calibration from the reference analytics of all samples from one field. The prediction is validated by LIBS data of the second field. The third method is a multivariate calibration approach based on partial least squares regression (PLSR). The LIBS spectra of the first field are used for training. Validation was carried out by 20-fold cross-validation using the LIBS data of the first field and independently on the second field data. The second univariate method yielded better calibration and prediction results compared to the first method, since matrix effects were better accounted for. PLSR did not strongly improve the prediction in comparison to the second univariate method.

## 1. Introduction

Precision agriculture (PA) requires reliable, affordable, immediately available soil data with sufficient spatial and temporal resolution [1]. Soil maps for PA are typically derived from soil sampling with subsequent laboratory analysis or from mapping with automated mobile proximal soil sensors. Soil sampling and laboratory analysis is time consuming and becomes prohibitively expensive if conducted on a fine grid [2]. As an alternative, mobile proximal soil sensors can measure several hundred points (different locations) per hectare [3,4,5,6,7]. However, most of the current soil sensors neither cover the whole range of soil fertility parameters nor do they directly measure them. The automated mobile proximal soil sensing systems frequently used in practical PA as well as research include geoelectrical, potentiometric pH, gamma-ray and spectral-optical sensors. Among these, pH electrodes most directly access a soil fertility parameter, namely active acidity [8]. Gamma-ray sensors detect radiation from K decay and from other isotopes. This can be correlated with K in clay minerals and with plant available K^+^ [9,10]. Spectral-optical sensors include spectrometers and multi-wavelength sensors in the visible and near-infrared region. Correlations with several soil fertility parameters were observed, particularly with organic matter [11]. In research, visible and near-infrared spectrometers were used to map several fertility parameters at the same time [12,13]. For practical applications, cheaper and more robust dual or multi-wavelength sensors for organic matter and soil moisture were commercialized (e.g., by Veris Technologies and Precision Planting). However, the relationships between optical soil properties and soil fertility parameters are variable due to large overlaps of absorption bands. Thus, optical online sensors require careful calibration for each field [11,14]. Geoelectric sensors traditionally formed the backbone of PA soil analysis efforts and they are still widely used due to their robust nature making them both dependable and suitable for field applications [15,16,17]. However, apparent soil electrical conductivity (ECa) is affected by many soil parameters, including water content, texture, salinity, bulk density and temperature [18]. Therefore, reference sampling in each field is required [19]. In a comparative study, Piiki et al. [20] recently addressed the issue of obtaining as much direct information with as little calibration as possible. In that study, X-ray fluorescence (XRF) spectroscopy produced the most reliable predictions of soil parameters due to its direct detection of elemental compositions. XRF has gained interest in recent years due to the availability of handheld sensors [21,22,23]. The drawbacks of XRF include the harmful X-rays, long measurement times and the restriction to heavier elements.

Laser-induced breakdown spectroscopy (LIBS) is a promising alternative to XRF for determining element mass fractions in soils. In this method, an intense pulse of laser radiation is focused onto the soil, where it ablates material from the surface and creates a microplasma. Subsequently, excited atoms and ions in the plasma emit specific radiation which can be analyzed to elucidate the elemental composition of the sample [24,25,26,27,28]. Ablation and plasma excitation are both highly complex phenomena. Since the interaction of the laser radiation with the sample surface is influenced by its composition and structure, a matrix-dependence of the signal response is observed. Matrix effects result from the light-to-sample coupling, collisional interactions within the plasma, and plasma temperature, among others, all of which influence the ratio of neutral and ionized species and self-absorption. These matrix effects and spectral interferences were already investigated in different types of soil [29,30]. In LIBS soil analyses, the whole spectrum of elements can be accessible. Depending on the calibration effort, elements can be determined qualitatively or quantitatively. This allows the direct analysis of macro and micro nutrients since no or only minimal sample preparation is necessary. In order to achieve the power density required for plasma generation, the laser light pulse, of typically nanosecond duration, is usually focused to a spot of about 10–500 µm diameter. As a consequence, the soil micro-heterogeneity has to be considered in order to obtain representative results. This can be achieved by averaging multiple spectra.

Additional advantages of LIBS include the measurement speed, safety, as well as the portability of the technique. These attributes make the method particularly interesting for on-site soil mapping. While large-scale soil mapping applications of LIBS have not been reported yet and early LIBS investigations of soils were focused on pollutants [31], the detection of nutrients in soils was already demonstrated in some publications. Diaz et al. [32] were able to determine detection limits of P, Fe, Mg, Ca and Na by univariate calibration in fertilizer/soil mixtures. Yongcheng et al. [33] could improve the prediction of the Mg mass fraction by using a multivariate regression model that incorporates the lines of other metals present in the soil. Nicolodelli et al. [34] investigated the feasibility of measuring C in soils with a low resolution spectrometer. Rühlmann et al. [35] compare univariate and multivariate data evaluation approaches for the quantification of the nutrient Ca in reference soils. A review of recent work on the application of LIBS for investigating agricultural materials can be found in [36].

In this work, LIBS spectra of soils were measured in the laboratory as a fundamental study and to provide a basis for the future application of LIBS directly on agricultural fields. The aim of the study was the evaluation of different quantification approaches for LIBS data which consider the matrix effect. Another goal was to explore how a calibration obtained for one field can be transferred to another one. The accuracy of standard-free LIBS approaches [31,37] for the analysis of the very complex matrix soil is still unsatisfactory. Therefore, the focus of this work was to compare different univariate or multivariate calibration methods for the determination of nutrient mass fractions. The target parameters of the investigation were the major nutrients Ca and Mg as well as the minor nutrient Fe. One aim of this work was to examine whether the quantification of nutrients on the size scale of a field can be carried out by univariate calibration or whether a multivariate method, namely partial least squares regression (PLSR) has to be applied. Different calibration strategies, such as the generation of calibration standards by standard addition to a single reference soil sample and the use of multiple reference soil samples, were compared. The heterogeneity of the soil samples was characterized by principal component analysis (PCA). The plasma was generated using UV radiation (355 nm), which was recommended for soil investigations [30] in contrast to the widely applied NIR radiation (1064 nm).

## 2. Materials and Methods

**Soil samples and reference analysis.** A total of 139 samples from two agricultural fields near the village of Wilmersdorf in Northeast Germany (53°06′ N, 13°54′ E) were investigated. The regional soilscape was formed by the last glaciation about 10,000 years ago and the following postglacial processes. The parent material of the soil consists of calcareous glacial till covered by sandy deposits. Soil texture varies between sand, loamy sand, and sandy loam in the topsoil. The main soil types are alfisols. The samples were collected in 2011 to obtain reference data for proximal soil sensing as published by Schirrmann, Gebbers and Kramer [14]. Reference analysis for elements was carried out by inductively coupled plasma optical emission spectroscopy (ICP-OES) after aqua regia extraction by a certified laboratory. As target parameters, Ca and Mg were selected as examples for major nutrients and Fe as an example for minor nutrients.

**ECa mapping**: ECa [mS/m] was mapped with a Veris 3100 system (Veris Technologies, Salina, KS, USA) in 2011. Only data from the shallow measurement were used. Data were interpolated by block kriging (a) on a regular grid for visualization and (b) on the sampling locations for correlation analysis.

**LIBS setup and measurement parameters.** The plasma was created using a Nd:YAG laser (Quanta-Ray, Spectra-Physics, Santa Clara, CA, USA, *λ* = 355 nm, *E* = 90 mJ). Emissions were collected by a concave mirror, coupled into an optical fiber and guided to an echelle spectrometer (Aryelle Butterfly, LTB, Berlin, Germany) equipped with an ICCD camera (iStar, AndorTechnology, Belfast, UK). The spectrometer has two separate wavelength ranges (UV range: 190–330 nm, VIS range: 275–750 nm) and a resolution of 20–30 pm. A total of 200 single shot spectra were recorded per sample in the UV as well as in the VIS range. The sample holder was rotated and linearly translated during measurements forming a spiral-like trace of ablation events. Optimization of LIBS spectra led to the following measurement parameters: a detection delay of 2 μs, a measurement window of 10 μs as well as a constant amplification factor of the iCCD camera.

**Sample treatment for LIBS.** The soil samples were mixed with starch (19 wt% final mass fraction), ground in an agate ball mill and subsequently pressed into pellets. One pellet was created for each field sample point. For standard addition, the sample point of the first field with the lowest intensity of the respective element peak was chosen for each investigated element. To that end, the soils were mixed with the respective amounts of target elements added as salts (CaCO_3_, MgCl_2_ and FeS, the amount of starch was reduced in order to keep soil fraction constant) and also formed into pellets. The salts were obtained from Sigma Aldrich (St. Louis, MO, USA).

**Data pretreatment.** Pretreatment of the LIBS spectra consisted of outlier (e.g., spectra where the laser did not fire) removal using the following procedure. The total intensity of each spectrum for a given sample was calculated. The median of these values was derived and only the spectra in the range 0.75 × median ≤ total intensity ≤ 1.25 × median were averaged to yield a single spectrum for each soil sample. For PLSR calibration (third method, see below) spectra were mean centered. Logarithms of the known element mass fractions were used as y variables when the data distribution was strongly skewed. For univariate calibration, the peaks of investigated elements were integrated for each averaged spectrum individually. For multivariate analysis, the entire spectra of either the UV or the VIS region were used.

**Calibration.***Standard addition univariate calibration.* The first calibration method was based on standard addition using just one soil sample and applying the derived univariate calibration model to the LIBS data of both fields. Using the standard addition method potentially has several advantages. First, the technique can be used to determine the mass fraction of one or several nutrients in one soil sample by extrapolating the mass fraction of the base sample from the samples with added known quantities of an element. Second, as applied in this work, reference analysis of the pure soil sample by the traditional digestion method and in combination with samples where increasing amounts of the nutrients were added can provide LIBS calibration curves. One additional benefit is that standard addition yields response curves where the matrix effect of the local soil type is accounted for. The nutrients are advantageously added as salts. However, care must be taken that the salts are not hygroscopic. The third advantage is that the large range of nutrient mass fractions covered for the determination of calibration curves facilitates assigning lines to elements and finding the best lines to use for the quantification of each element. Potentially, different lines could be chosen for different mass fraction ranges. The most intense lines might be useful at low element mass fractions, while at high element mass fractions, these intense lines can be self-absorbed. Therefore, choosing weaker lines can become beneficial.

*Reference univariate calibration*. The second method derived a univariate calibration model from the reference analytics of all samples from one field. The prediction is validated using the second field. This approach accounts for the matrix effects even better, as the variation of the matrix across a field is also reflected. Furthermore, the error in the laboratory-based reference analytics of one soil sample has a large influence on the LIBS-based results. Therefore, a larger data pool with known reference values and a broader variety of matrices should be regarded. In our case, the data of the first field is used for the calibration and the data of the second field is used for the validation. The concept of this procedure is that reference data gained in one year could be used to build a calibration for a specific field. In subsequent years, it will not be necessary to take new samples and the calibration can therefore also be applied to fields in the near surrounding and to fields with a similar soil type. Furthermore, a much larger number of data points can be evaluated, as LIBS can be employed directly on the field, allowing a closer spatial mapping of the fields.

*Reference multivariate calibration*. The third approach was a multivariate calibration based on PLSR. PLSR was done using the kernel algorithm and a maximum of 7 components. The LIBS spectra of the first field were used for training. Validation was carried out by 20-fold cross-validation using the data of the first field and independently by testing the model trained on the data of the first field with the data of the second field.

**Software**: Origin (OriginLab, Northampton, MA, USA) was used for PCA. Unscrambler X (Camo Analytics, Oslo, Norway) was used to perform PLSR.

## 3. Results and Discussion

### 3.1. LIBS Spectra

**Figure 1:**
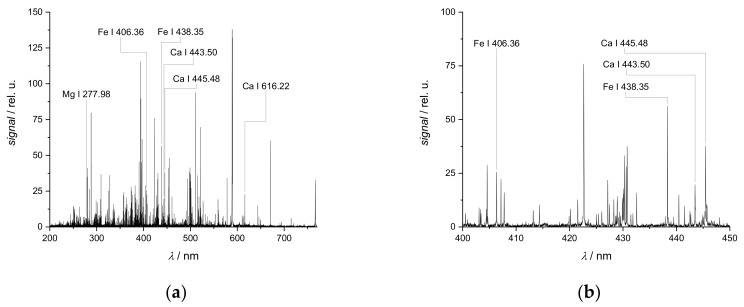
Typical soil laser-induced breakdown spectroscopy (LIBS) spectrum (composite of UV and VIS part of the LIBS spectrum), (**a**) full wavelength range, (**b**) detail view of wavelength range 400–450 nm.

LIBS spectra of soils are rich in lines, mostly due to the presence of Fe and other transition metals (Figure 1). Additionally, lines of most minor and major mineral nutrients are found, the lines of metal nutrients are particularly intense.

But the plethora of lines complicates their assignment to elements, as peak overlapping is frequently observed. The line identification is not straightforward as the total elemental composition of soils is generally not known. Otherwise, the limitation of the measurement campaign on one or two fields is advantageous for peak assignment as the soil heterogeneity is not as great as on a larger scale. Most peaks are generally found in spectra of all samples (different points on the field) but their intensities still differ substantially on the scale of one field. This observation is due to variations of the element mass fractions across the field, which is also the origin of the strong matrix effects encountered and necessitates the determination of element distributions in precision agriculture.

### 3.2. Standard Addition Calibration

**Figure 2:**
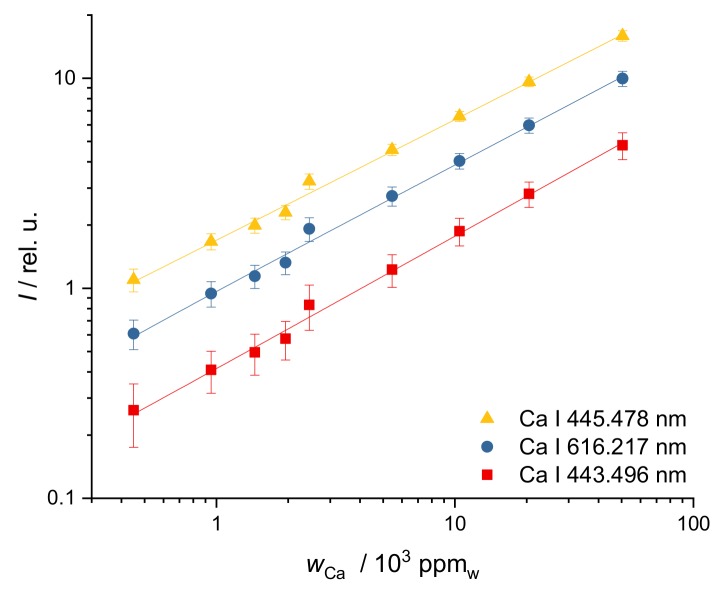
Calibration curves of different Ca lines in soil, integrated line intensity over calculated Ca mass fraction: R^2^ (443.496 nm) = 0.994, R^2^ (445.478 nm) = 0.996, R^2^ (616.217 nm) = 0.993 (individual wavelengths are offset for visibility).

Ca mass fractions vary greatly (from 500 ppm_w_ to 5 wt%) in the investigated soils. A corresponding calibration curve has to reflect a mass fraction range of nearly three orders of magnitude and is best presented in a double-logarithmic diagram. The fitting function *I* = a *w*^c^, where *I* is line intensity, *w* the mass fraction and a and c are the optimized constants, essentially equivalent to taking the logarithm of the data prior to linear fitting, was used. The calibration curves for three different Ca lines (at 443.496 nm, 445.478 nm, 616.217 nm) are shown in Figure 2. These lines provided best results in terms of repeatability (precision), linearity (R^2^ > 0.993) and usability over the entire range of relevant mass fractions.

**Figure 3:**
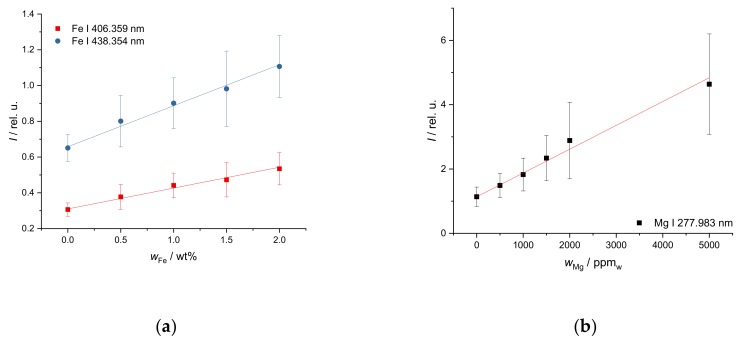
Response curve of (**a**) FeS and (**b**) MgCl_2_ in soil, integrated line intensity over calculated (**a**) Fe and (**b**) Mg mass fractions, (**a**) R^2^ (406.359 nm) = 0.988, R^2^ (438.354 nm) = 0.992, (**b**) R^2^ (277.983 nm) = 0.989.

The Fe mass fraction in the investigated soil (from 0.5 wt% to 6 wt%) is on a higher mass fraction level but covers a narrower range than the Ca mass fraction. Thus, a linear calibration plot was selected (Figure 3a). The LIBS spectrum contains a very high number of Fe lines. Due to the high mass fraction, many of them show strong effects of self-absorption. This requires a careful selection of lines. The lines at 406.359 nm and 438.354 nm (see Figure 3a) are best suited for calibration and yield R^2^ > 0.988 for λ = 406.359 nm and R^2^ > 0.992 for λ = 438.354 nm. The repeatability is not as good as for Ca as the error bars show, but quantification is still possible.

Mg appears in the soils at low to intermediate mass fractions (from 700 ppm_w_ to 4000 ppm_w_) and covers a narrow mass fraction range. Therefore, a linear calibration curve was selected as well. Useful Mg lines are only found in the UV region. The repeatability is worse at higher mass fractions as evidenced by higher error bars. The line at 277.983 nm shown in Figure 3b yields the best result in terms of linearity over a range of mass fractions (R^2^ = 0.989).

For all chosen elements, lines could be found in the available spectral range that are free from overlap, less prone to self-absorption effects and provide good dynamic ranges. The detection limits usually are in the range of 100 ppm which is adequate for the soils in question. Lower detection limits would only be needed for micronutrients.

### 3.3. Application of Standard Addition Calibration to the First Field

**Figure 4:**
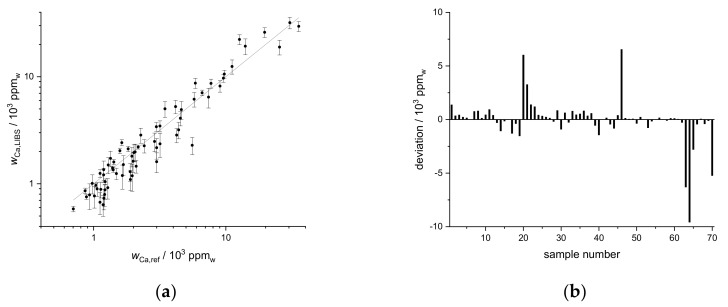
(**a**) Mass fraction of Ca predicted by LIBS univariate calibration based on standard addition (*w*_Ca,LIBS_) compared to reference values obtained by ICP-OES upon aqua regia extraction (*w*_Ca,ref_) for the first field, R^2^ = 0.91, (**b**) deviation of predicted from reference value sorted by sample number.

In order to obtain the mass fraction distribution of the nutrients on a field, the standard addition calibration was applied to LIBS spectra of all samples (points on the field) for predicting the element mass fractions. A comparison of the values thus obtained from the LIBS measurements and the reference values obtained by ICP-OES yields insights into the performance of the calibration method. This is shown for the nutrient Ca in Figure 4a, where the Ca mass fractions measured by LIBS are the averages of the results for the three lines in the LIBS spectrum. The line in Figure 4a presents the identity of values obtained by LIBS and ICP-OES. The Ca mass fractions obtained by LIBS scatter around this line and show a very good agreement over the range of mass fractions investigated. Thus, a univariate calibration, built using only one reference sample, can be used for predicting the Ca mass fraction for the entire field. Absolute deviations for the different sample points on the field are shown in Figure 4b with the highest errors in the range of 1%. The deviations seem accumulated around certain points of the field as sample numbers indicate proximity. Because samples were taken along predefined lines across the field, this could be an indication of different soil types and thus matrix effects at those positions.

**Figure 5:**
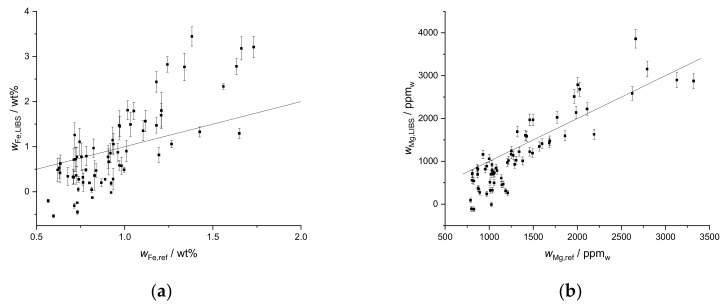
Mass fractions of (**a**) Fe and (**b**) Mg predicted by LIBS univariate calibration based on standard addition compared to reference values obtained by ICP-OES upon aqua regia extraction for the first field.

Results were not as promising for Fe (Figure 5a). Although a rough agreement is present in values around 1 wt%, lower and especially higher Fe mass fractions are overestimated by as much as twice the reference value. Reasons could be the relatively narrow range of mass fractions and especially the high overall Fe mass fractions, leading to e.g., self-absorption. The results of the Mg prediction are shown in Figure 5b. Agreement of the results is much better, although in the low mass fraction region, a larger spread of the data points is observed. Overall, this kind of calibration is satisfactory for the major nutrients Ca and Mg but the predictive power is limited for the minor nutrient Fe.

### 3.4. Application of Standard Addition Calibration to Second Field

As a further validation, the same calibration curves were also applied to the second field data, which does not contain the soil sample used for obtaining the standard addition calibration. Therefore, the results of this second field are a measure of the predictive power for local agriculturally-used areas in the surrounding of the calibration field. It is likely that the soil types on these fields will have a similar characteristic.

**Figure 6:**
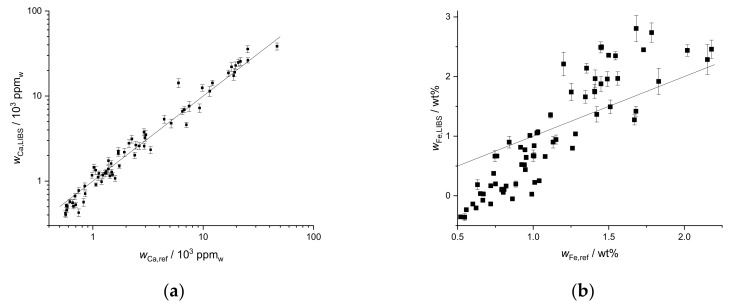
Mass fraction of (**a**) Ca and (**b**) Fe predicted by LIBS univariate calibration based on standard addition compared to reference values obtained by ICP-OES upon aqua regia extraction for the second field, R^2^ (Ca) = 0.60.

**Figure 7:**
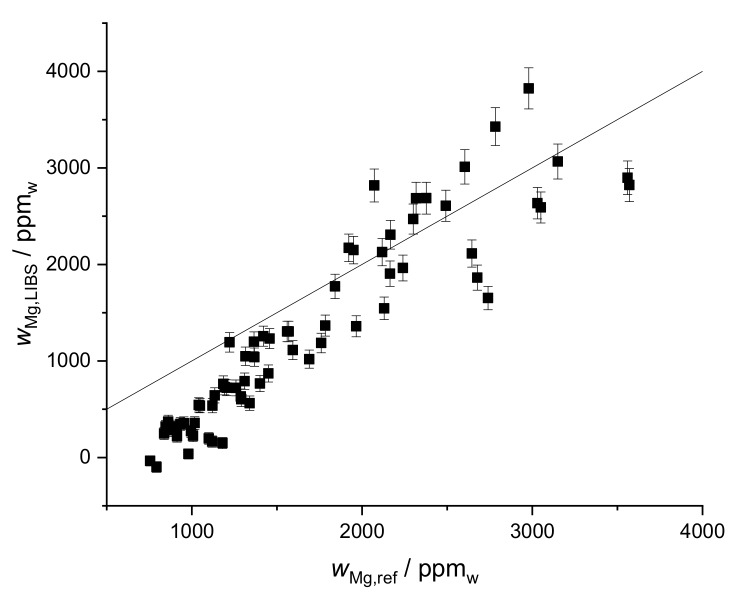
Mass fraction of Mg predicted by LIBS univariate calibration based on standard addition compared to reference values obtained by ICP-OES upon aqua regia extraction for the second field.

The prediction results of the three elements for both fields are similar (Figure 6 and Figure 7). The best agreements are again achieved for Ca and Mg, especially the correlation for Ca is excellent. The prediction for Ca seems to be robust and can be also used for different fields of similar soil types. The deviation of the Mg prediction (Figure 7) is a bit stronger than for the first field. While it could still be usable in practice to get a rough estimation of the Mg mass fraction, a better method is desirable. The agreement of the values of the second field in the Fe plot (Figure 6b) is actually better than for the first field from which the soil for the calibration was taken, although the range of mass fractions is larger. However, the deviation is still present, and the calibration can only be used as a rough estimation.

Although the calibration method established from one sample works fine for the element Ca, even on different fields, the applicability to other nutrients is limited. The most important reason for this finding is the matrix effect, which is not sufficiently taken into account by this method. On a future field campaign, the discussed peaks in the LIBS spectra can, however, be used to find points of extreme element mass fractions, which are then crucial sampling points from which a better calibration can be built.

### 3.5. Reference Univariate Calibration

**Figure 8:**
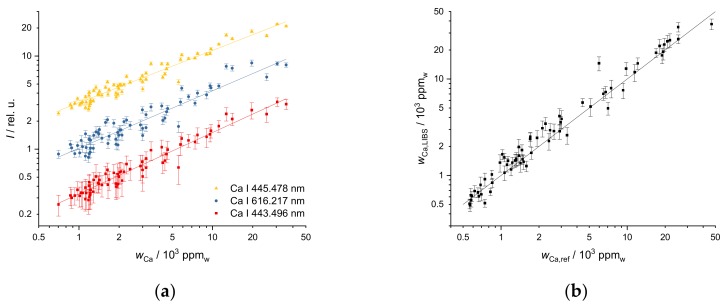
(**a**) Univariate calibration of Ca mass fraction calculated for data of the first field, R^2^ (443.496 nm) = 0.93, R^2^ (445.478 nm) = 0.94, R^2^ (616.217 nm) = 0.86 (individual wavelengths are offset for visibility), (**b**) Calibration applied to the second field, R^2^ = 0.93.

The results of the reference univariate calibration using all samples from the first field and its application to the second field are shown below. The calibration data of the LIBS response for three different wavelengths as a function of the Ca mass fraction obtained by the reference analysis are shown in Figure 8a. For all wavelengths, a good regression (e.g., R^2^ (445.478 nm) = 0.94) between data of the LIBS and reference analytical method is obtained, the coefficients of determination for the lines at 443.496 nm and 445.478 nm are better than those for the line at 616.217 nm. The calibration curves are applied to the LIBS data of the second field (Figure 8b), the Ca mass reported as measured by LIBS are the averages of the results for the three lines. This procedure results in predicted values that are in very good agreement with the reference data (R^2^ = 0.93).

**Figure 9:**
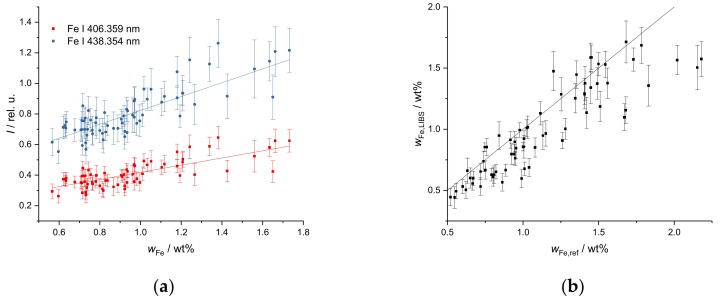
(**a**) Univariate calibration of Fe mass fraction calculated for data of the first field, R^2^ (406.359 nm) = 0.53, R^2^ (438.354 nm) = 0.61; (**b**) Calibration applied to the second field, R^2^ = 0.51, without two most obvious outliers (same samples as for Ca) R^2^ = 0.68.

The same procedure was applied to Fe (Figure 9). The calibration plots (Figure 9a) took two signals at the wavelengths at 406.359 nm and 438.354 nm into account but yielded a poorer prediction than that found for Ca. However, the larger amount of data used for the calibration compared to the standard addition method results in a better prediction for the second field (Figure 9b). Here, the matrix effects are better accounted for. Although the result is acceptable, a univariate calibration might not be ideal for Fe.

**Figure 10:**
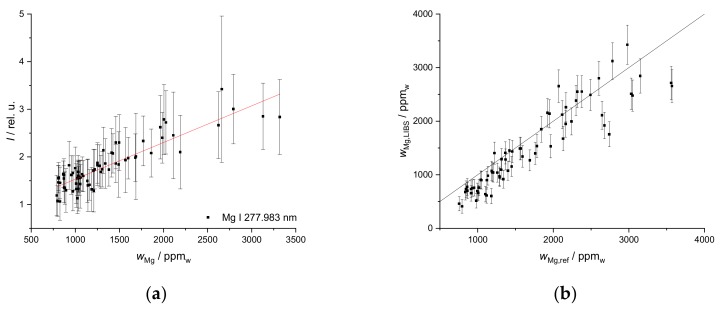
(**a**) Univariate calibration of Mg mass fraction calculated for data of the first field, the line used is Mg I 277.983 nm, R^2^ = 0.71, (**b**) Calibration applied to the second field, R^2^ = 0.51, without two most obvious outliers (same samples as for Ca) R^2^ = 0.76.

While the calibration plot for Mg is characterized by a linear relationship but relatively large error bars (Figure 10a), the validation plot (Figure 10b) obtained on the second field actually has a better coefficient of determination than the calibration fit itself. Although this can be explained by a few high error intervals of the intensities used for the calibration, it is still a promising result.

Overall, it can be concluded that using actual field samples for building the calibration improves the performance for the univariate calibration. Such univariate methods can thus be used for predicting values. In the future, online LIBS measurements on the field can be used to determine which samples to use for laboratory analyses. If these samples represent a good range of the values of interest, a strong calibration can be built, which can in turn be used to predict values for all points of the online measurements.

### 3.6. Multivariate Analysis

**Figure 11:**
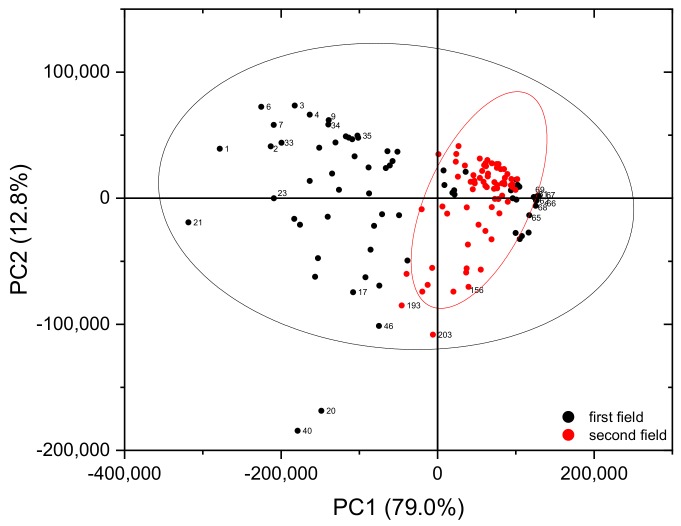
PCA of all LIBS spectra of the first (black points) and the second field (red points).

One possibility for characterizing the heterogeneity of soil compositions of the individual fields, and of the first field in relation to the second field, is to apply PCA. For that purpose, the complete LIBS spectra were introduced into PCA. In the score plot (Figure 11) the first two components, which explain 93% of the variance, are presented. Grouping the data points by fields implies that the heterogeneity of the second field (red points) is lower than that of the first (black points). This also explains why the univariate calibrations were largely successful and justifies using the first field as training data and the second field as validation data in multivariate analyses.

**Figure 12:**
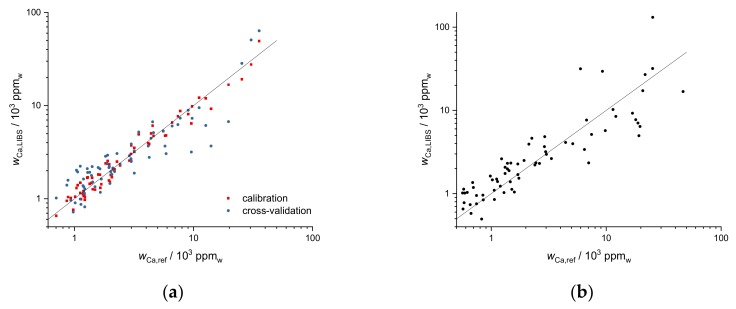
(**a**) Partial least squares regression (PLSR) of Ca data for the first field (UV range), red squares represent calibration data (R^2^ = 0.96), blue points represent 20-fold cross-validation (R^2^ = 0.83), (**b**) application of PLSR of Ca data to the second field (R^2^ = 0.58).

As a multivariate regression method, PLSR was performed on our data. Validation of the results of the regression model was taken into account in two different ways. First, 20-fold cross-validation was applied on the data of the first field. This is demonstrated for Ca data of the first field in Figure 12a. Correlation plots of Ca mass fractions predicted by LIBS and by ICP-OES are shown. The red squares are the result of the PLSR calibration, and the blue points represent 20-fold cross-validation. For PLSR, the logarithms of Ca values were taken, and 5 components were used.

The PLSR cross-validation data (R^2^ = 0.83) demonstrate the potential of multivariate regression for predicting mass fractions on the same field. A second, additional validation is obtained by applying the calibration obtained of the first field to the second field, demonstrating how universal a calibration could become. This is shown in Figure 12b for Ca and gives an impression of the prediction quality of PLSR calibrations for additional fields with similar soil types (R^2^ = 0.58).

**Figure 13:**
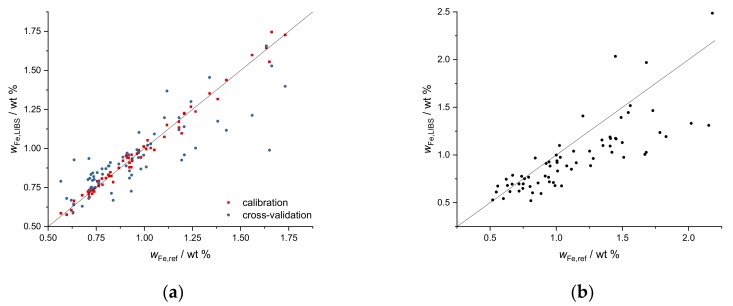

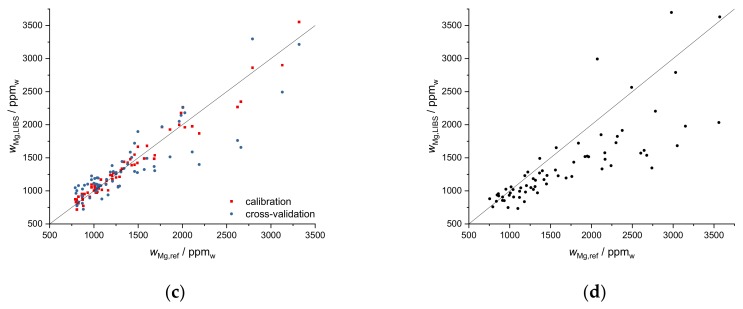
(**a**) PLSR of Fe data for the first field (UV range), red points represent calibration data (R^2^ = 0.986), blue diamonds represent 20-fold cross-validation (R^2^ = 0.70), (**b**) application of PLSR of Fe data to the second field R^2^ = 0.37, (**c**) PLSR of Mg data for the first field (UV range), red points represent calibration data (R^2^ = 0.96), blue diamonds represent 20-fold cross-validation R^2^ = 0.79, (**d**) application of PLSR of Fe data to the second field R^2^ = 0.38.

PLSR was also applied to the elements Fe and Mg (Figure 13). For both elements, the logarithms of the mass fractions were used for PLSR. Seven components were used for PLSR of the Fe data, and 6 components were used for PLSR of the Mg data. The cross-validation plots of both elements scatter a bit stronger than the Ca plot (R^2^ (Fe) = 0.70, R^2^ (Mg) = 0.79). The application of the calibration data of the first to the second field results in a similar scattering of the predicted values. With increasing element mass fractions, the deviation of predicted values from reference values becomes systematically larger. LIBS underestimates high Fe and Mg mass fractions.

### 3.7. Field Maps

**Figure 14:**
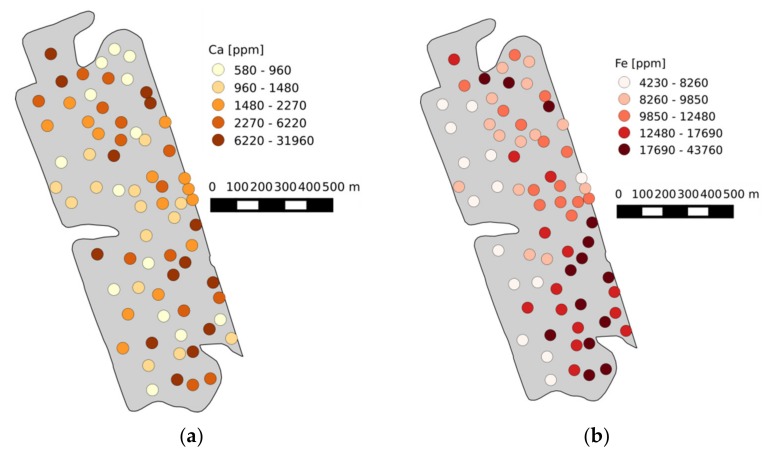

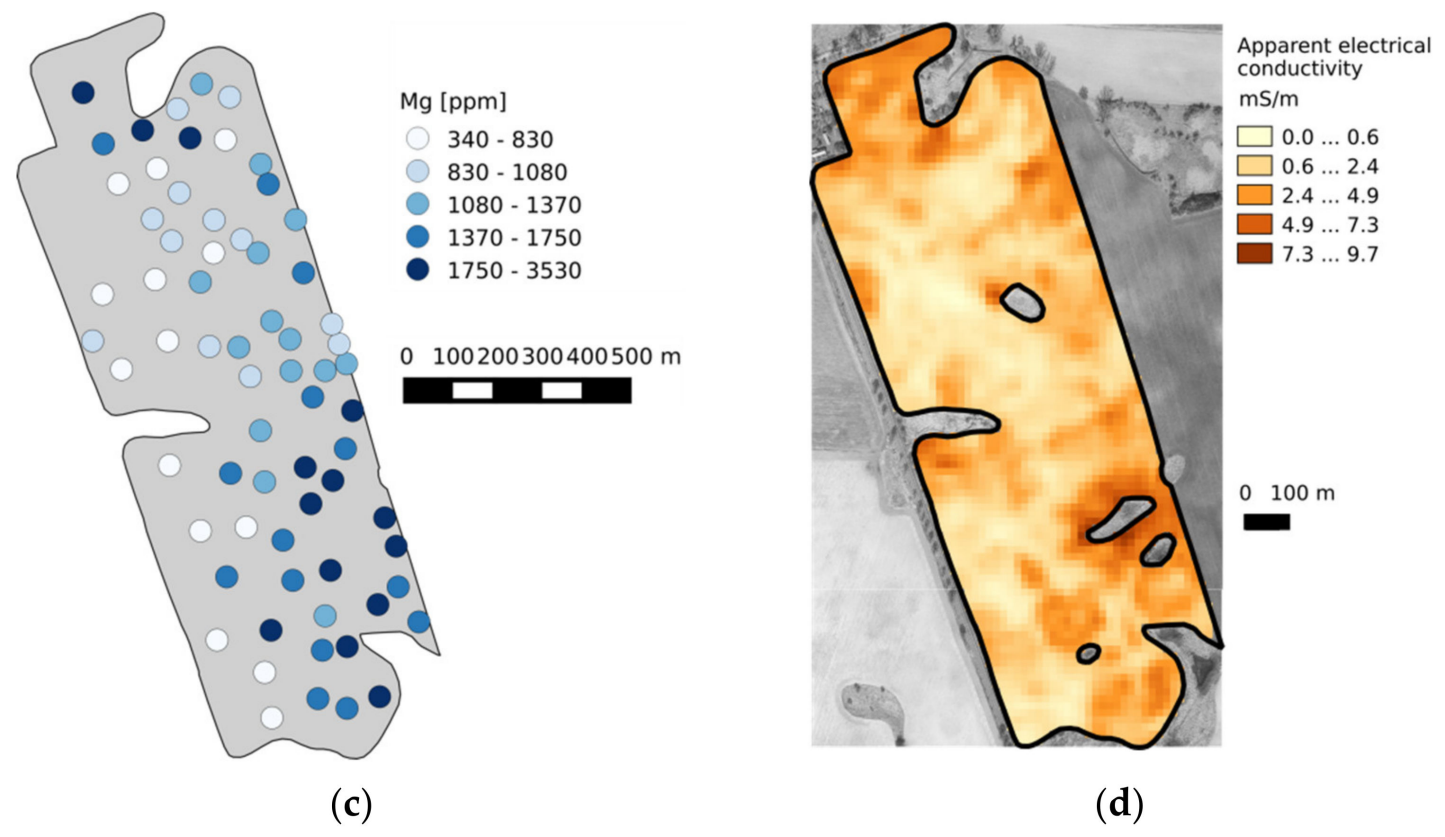
Maps showing the element distribution of (**a**) Ca, (**b**) Fe, (**c**) Mg and (**d**) ECa on the first field (north is up).

One main aim in precision farming is the mapping of nutrient distributions on agricultural fields. In connection with fertilizer recommendations based on measured data as well as models, these maps help the farmers identify nutrient-depleted areas. The change between nutrient-rich and depleted areas can be seen on the three maps of Figure 14. Thus, the eastern areas are richer in all three elements while the western areas are depleted.

This spatial trend was further explained by the evaluation of the ECa map (Figure 14d), which was recorded directly on the field. The ECa values were positively correlated with all elements as determined by the reference method and by LIBS.

## 4. Summary and Conclusions

LIBS is a promising method for efficient soil data collection at high spatial resolution, as required for precision agriculture. This is due to the capability of LIBS to rapidly measure mass fractions of many elements simultaneously without or only very little sample preparation. These advantages are contrasted by a strong matrix dependence of the LIBS signal which requires careful calibration and data evaluation methods.

In this work, different approaches for the evaluation of LIBS data were investigated. The first univariate method was based on standard addition to just one soil sample, the second was a univariate method based on a larger number of samples characterized by reference analytics. This second method accounted for matrix effects and thus yielded better predictions. The third method was a multivariate method (PLSR) which yielded better calibration curves, but did not substantially improve the prediction compared to the reference univariate method. Alternative and maybe better suited multivariate methods can potentially provide better results.

The work begun here should be continued by expanding the LIBS measurements to a larger database, containing more and especially different soil types. It could also be interesting to obtain soil samples from the same field in different years and verify if the same calibrations can be used. The classification of soil samples based on different soil types and the application of tailored calibration methods for these soil types can improve the accuracy and repeatability of measuring results in the future. Additionally, the amount of sample preparation performed in this work has to be reduced in order to transfer the method to the field.
